# Hybrid Multi-Infeed Receiver Line Longitudinal Protection Scheme Based on Voltage Waveform Comprehensive Distance Similarity

**DOI:** 10.3390/s24051601

**Published:** 2024-02-29

**Authors:** Shuping Gao, Xiaofang Li, Guobing Song, Han Zheng, Yunqing Duan

**Affiliations:** 1 Department of Electrical and Control Engineering, Xi’an University of Science and Technology, Xi’an 710054, China; gao.sp2003@163.com (S.G.); zh15249242959@163.com (H.Z.); dyq1909799547@163.com (Y.D.); 2 Xi’an Key Laboratory of Electrical Equipment Condition Monitoring and Power Supply Security, Xi’an 710054, China; 3 Department of Electrical Engineering, Xi’an Jiaotong University, Xi’an 710049, China; song.gb@mail.xjtu.edu.cn

**Keywords:** hybrid DC multi-feed system, pilot protection, voltage waveform, comprehensive distance similarity

## Abstract

The coupling of AC and DC power will impact the protective actions on the AC side and pose a threat to the stable operation of the interconnection system. Therefore, a new longitudinal protection method is proposed based on the comprehensive distance similarity of voltage waveforms. Initially, the measured voltage and current data are extracted to calculate the reference voltage, and the voltage waveform fitting is optimized. Subsequently, the Euclidean dynamic time warp (DTW) distance and entropy weight method are utilized to process the voltage waveform, enabling the calculation of its comprehensive distance similarity. This similarity is adopted to determine fault location. A hybrid DC multi-feed AC/DC interconnection system, incorporating a line commutated converter-voltage source converter (LCC-VSC) and a line commutated converter-modular multilevel converter (LCC-MMC), was established in PSCAD, and fault data were simulated and output. The effectiveness of the protection scheme was validated using MATLAB. Simulation results demonstrate that the proposed method can accurately distinguish between faults inside and outside a region. When compared to existing protection methods, it demonstrates superior performance in resisting transition resistance and noise interference, while also mitigating the impact of data asynchronicity. The speed and reliability of the method are further enhanced.

## 1. Introduction

With the continuous development of China’s socio-economic landscape, the electrical demand is rapidly increasing. In order to balance the rising demand for electricity and resource allocation, China is accelerating the development of the strategies of “west to east power transmission” and “north to south power transmission”. High voltage direct current (HVDC) transmission technology is being utilized in grid development to meet the inevitable requirements of long distances, high capacity, and high voltage levels [1,2].

A line commutated converter (LCC) is suitable for long-distance and high-capacity scenarios. However, due to its semiconductor control, it suffers from the drawback of commutation failure and is generally used at the rectifier end. Conversely, a voltage source converter (VSC), employing fully controlled devices, can resist commutation failure on the inverter side and flexibly control the active and reactive power of the system. Its application in multi-feed systems can enhance operational flexibility at the receiving end and expand transmission capacity [3,4]. However, the application of VSC as a fully controlled device means a higher economic investment. Therefore, the combination of LCC and VSC in multi-infeed DC transmission harnesses the advantages of both to compensate for their respective shortcomings. This becomes especially advantageous in aspects such as multi-AC grid interconnection, multiple power supply, and multiple load connection, fully leveraging the economic and flexible nature of DC transmission. However, the introduction of multi-DC lines for feed-in complicates the system and makes protection prone to false operations [5]. Hence, research is necessary for the relay protection of the receiving end AC system.

Currently, the protection methods for AC transmission lines primarily rely on traditional relay protection principles, including directional protection, distance protection, and differential protection [6]. In comparison to traditional AC transmission, the fault characteristics for AC lines in hybrid multi-infeed DC systems change, prompting scholars to research the applicability of traditional relay protection [7,8,9,10,11,12,13]. For instance, Hao et al. [7] discovered that due to the fact that the protection unit near the inverter side can only identify continuous commutation failures occurring after a fault, it is ineffective at identifying faults where there is no commutation failure after the fault or when there is only one commutation failure. Therefore, when a single transmission corridor structure is used on the AC output side of the inverter side, traditional directional protection is not suitable for AC/DC hybrid interconnected networks. Additionally, continuous commutation failures might lead to severe faults on the AC side of multi-infeed DC systems, causing a sharp increase in the reactive power demand for system fault recovery [7,8,9,10]. This can lead to transient power reversals in neighboring multiple AC lines and the DC system [11,12], while the reverse power flow can lead to potential misoperation of the longitudinal directional protection [13]. Meanwhile, Huang et al. [14] proposed improvements to the protection setting principles of the second zone of distance protection based on fault characteristics but were unable to address errors caused by high-order harmonics. Additionally, Huang et al. [15] presented a rapid fault detection method based on instantaneous active power and fault transient components to alleviate the effects of commutation failures and reactive power compensation. Ma et al. [16] used time-domain differential equations to calculate fault point location, enhancing the protective performance of fault distance measurement, but with poor anti-interference capabilities that are unable to protect the entire length of the line. Moreover, Zheng et al. [17] analyzed the impact of non-periodic components and harmonic components caused by DC feeding on the accuracy of algorithms, resulting in decreased protective operation performance. In summary, the multi-DC system feeding complicates the research for AC line protection at the receiving end of hybrid AC/DC systems, currently highlighting the following limitations: (1) Traditional directional and distance protection strategies are challenging to ensure the reliability of AC protection, and existing protection schemes still have shortcomings. (2) Higher harmonics on the DC side can also affect the reliability of AC protection operations. (3) Currently, there is limited research and analysis on the protection at the AC receiving end, with most of it being qualitative analysis.

This article introduces a new protection principle based on comprehensive voltage waveform analysis for multi-infeed AC/DC hybrid interconnected systems. The key contributions are as follows:

(1) A novel protection method is proposed that relies on the similarity of voltage waveforms between measuring and reference points. It addresses the issue of unreliable operation of AC protection in AC/DC hybrid systems.
(2) The Euclidean distance and dynamic time warping (DTW) distance are investigated to assess waveform similarity, thus avoiding the impact of data asynchrony or protection misoperation caused by a single distance metric.
(3) The least squares method (LSM) is employed to fit the voltage waveform, effectively mitigating interference from high-order harmonics.

The article is structured as follows: Section 2 introduces the measurement method for voltage waveform distance similarity. Section 3 proposes the topology of the hybrid AC/DC multi-infeed interconnected system and analyzes the system’s fault characteristics; it then describes the protection scheme steps and processes. In Section 4, a model is established in PSCAD, and MATLAB is used to simulate and verify the feasibility of the protection scheme. The conclusion is presented in Section 5.

## 2. Comprehensive Voltage Waveform Similarity Calculation

### 2.1. Waveform Processing

In the event of an AC fault in the AC/DC hybrid network, the fault phase voltage amplitude rapidly decreases. Additionally, as DC feed-in can bring about high-order harmonic interference, which will affect the electrical measurement of AC side, it is necessary to preprocess the waveforms of measured voltage and reference voltage in order to more visually reflect the trend of chang. For high-order harmonic interference, common engineering methods for treatment currently include wavelet transformation [18], Fast Fourier Transform (FFT), amplitude limiting filtering, median filtering, and other filtering methods. Wavelet transformation can provide high-resolution time-frequency analysis, but the selection of wavelet basis and the setting of decomposition layers may be complex, and the computational complexity is high, increasing processing time and resource consumption. Fast Fourier Transform (FFT) has significant advantages in computational efficiency, but its performance for abrupt and non-stable signals is poor, and its processing capacity for small sample signals is limited, making it limited in application to relay protection methods. Amplitude-limiting filtering and median filtering have poor treatment effects for periodic interferences in AC/DC power grids and are prone to eliminating important waveform information [19]. Therefore, waveform signal processing must ensure both rapid response and the retention of waveform information that reflects trends. In this study, the use of the LSM to fit voltage waveforms meets both of these requirements.

LSM is a mathematical fitting technique that connects discrete points on a plane through a curve. It generally assumes that the curve can be represented by the following:

(1) $$f\left(x\right)=a_{0}+a_{1}x+a_{2}x^{2}+a_{3}x^{3}+\cdots +a_{N}x^{b}$$

the unknown coefficients are denoted as *a*_0_, *a*_1_, *a*_2_…*a_N_*, and *b* in *x_b_* represents the power to which we want to fit.

Assuming the voltage waveform is $\left(x_{i},y_{i}\right)$, $i=\left(1,2,3,\cdots ,m\right)$, where *m* denotes the number of voltage values, the fitted curve is then given by the following:

(2) $$f^{\prime }\left(x\right)=\min\sum_{i}^{m}{\left[f^{\prime }\left(x_{i}\right)-y_{i}\right]}^{2}$$

By fitting the measured voltage and reference voltage waveforms, high-order harmonic interference can be eliminated while retaining the variation trend.

### 2.2. Euclidean Distance Calculation

Euclidean distance, also known as Euclidean metric, is commonly used to measure the true distance between two points in a space, reflecting the overall distribution characteristics of waveform similarity [20]. The instantaneous values of measured voltages at the protection installation points M and N are extracted. Corresponding reference voltages are calculated using Equation (1), defining the sample time sequences of the measured voltages and the reference voltages as *d_M_*, *d_N_*, *d_rM_*, and *d_rN_*, respectively. Here, $d_{M}=\left(x_{1},x_{2},x_{3},\cdots ,x_{n}\right)$, $d_{N}=\left(y_{1},y_{2},y_{3},\cdots ,y_{n}\right)$, $d_{rM}=\left(z_{1},z_{2},z_{3},\cdots ,z_{n}\right)$, $d_{rN}=\left(q_{1},q_{2},q_{3},\cdots ,q_{n}\right)$, and *n* represents the number of sample points, which was chosen based on the precision requirements of the protection method. The Euclidean distance of the voltage waveforms between the measured voltages and the corresponding reference voltages at the protection installation points on both sides is given by the following:

(3) $$D_{WM}\left(d_{M},d_{rM}\right)=\sqrt{\sum_{i=1}^{n}{\left(x_{i}-z_{i}\right)}^{2}}$$

(4) $$D_{WN}\left(d_{N},d_{rN}\right)=\sqrt{\sum_{i=1}^{n}{\left(y_{i}-q_{i}\right)}^{2}}$$

where *D_WM_* and *D_WN_* represent the Euclidean distance between the measured voltage and reference voltage waveforms at the installation points on sides M and N, respectively.

### 2.3. DTW Distance

The DTW method employs the concept of dynamic programming, adjusting the different sample points of sequences through compression and expansion, thus enabling a point in one sequence to match multiple points in another, optimizing the warped path. Therefore, when the DTW algorithm computes distance, it does not require a one-to-one correspondence of values based on the same sample points, unlike the calculation method for Euclidean distance. The DTW algorithm can address issues of sequence asynchrony, better capturing the overall dynamic characteristics of curves and measuring the overall similarity of shapes between sequences [21]. For the sampled sequences *d_M_* and *d_rM_*, an *n* × *m* distance matrix *D_M_* is constructed, where the elements are *D_M_*(*i*, *j*) as per Equation (5). Similarly, for the sampled sequences *d_N_* and *d_rN_*, an *n* × *m* distance matrix *D_N_* is built, with elements being *D_N_*(*i*, *j*) as per Equation (4).

(5) $$D_{M}\left(i,j\right)=\sqrt{{\left(x_{i}-z_{j}\right)}^{2}}\cdots 1\leq i\leq n,1\leq j\leq m$$

(6) $$D_{N}\left(i,j\right)=\sqrt{{\left(y_{i}-q_{j}\right)}^{2}}\cdots 1\leq i\leq n,1\leq j\leq m$$

where Equation (5) represents the Euclidean distance between the sequences (*d_M_*) and (*d_rM_*) at the sequence points (*x_i_*) and (*z_j_*), while Equation (6) represents the Euclidean distance between the sequences (*d_N_*) and (*d_rN_*) at the sequence points (*y_i_*) and (*q_j_*).

The set composed of each pair of adjacent elements from *D_M_*(*i*, *j*) and *D_N_*(*i*, *j*) is defined as the bent path of the adopted waveform, denoted by *L*. Taking path *L* as an example, the element *l_s_* refers to the coordinates of the *s*-th point on the path, i.e., *l_s_* = (*i*, *j*). Path *L* must satisfy the following two-part constraints:

(I) The selected path must include all sampling points.
(II) Each sample point needs to be matched with the adjacent sample point, such that if *l_s_* = (*i*, *j*), *l_s+_*_1_ = (*a*, *b*) satisfies $\left\{\begin{array}{c} 0\leq a-i\leq 1\\ 0\leq b-j\leq 1 \end{array}\right.$.

This yields the DTW distance between the sequences *d_M_* and *d_rM_*, and the DTW distance between the sequences *d_N_* and *d_rN_*:

(7) $$D_{Mdtw}\left(d_{M},d_{rM}\right)=\underset{L}{\min}\sum_{s=1}^{k}D\left(L_{s}\right)$$

(8) $$D_{Ndtw}\left(d_{N},d_{rN}\right)=\underset{R}{\min}\sum_{s=1}^{k}D\left(R_{s}\right)$$

where *D*(*L_s_*) and *D*(*R_s_*) represent the cumulative distance of the bent path *L* and *R*, while *D_Mdtw_* and *D_Ndtw_*, respectively, denote the waveform DTW distance between the measured voltage and the reference voltage at the installation locations of the protections on both sides.

### 2.4. Local Dynamic Mapping

In order to better describe the local dynamic characteristics of the voltage waveform, reflecting its trend information at each sampling interval and fully considering the accuracy of the algorithm, the discrete sampling sequences $d_{M}$, $d_{N}$, $d_{rM}$, and $d_{rN}$ are transformed into shape sequences of length ${d^{\prime }}_{M}$, ${d^{\prime }}_{N}$, ${d^{\prime }}_{rM}$, and ${d^{\prime }}_{rN}$. We take the element *x*′*_i_* as an example, as shown in Equation (9), where Δ*t* represents the time interval as follows:

(9) $$x_{i}^{\prime }=\frac{x_{i+1}-x_{i}}{\Delta t}\cdots \cdots i=1,2,\cdots ,n-1$$

### 2.5. Comprehensive Voltage Waveform Similarity Description

This chapter combines the Euclidean distance with the DTW distance to propose a measurement method for the comprehensive voltage waveform similarity that encompasses overall, local, and dynamic characteristics.

(10) $$D_{M\varphi }=\alpha D_{WM}\left(d_{M},d_{rM}\right)+\beta D_{Mdtw}\left(d_{M},d_{rM}\right)+\gamma D_{Mdtw}\left(d_{M}^{\prime },d_{rM}^{\prime }\right)$$

(11) $$D_{N\varphi }=\alpha D_{WN}\left(d_{N},d_{rN}\right)+\beta D_{Ndtw}\left(d_{N},d_{rN}\right)+\gamma D_{Ndtw}\left(d_{N}^{\prime },d_{rN}^{\prime }\right)$$

where *D_M__φ_* and *D_N__φ_* represent the comprehensive distance similarity of the measured voltage and reference voltage waveforms at the protection installation points on M and N sides, where *φ* = A, B, C represents the phase sequence index. The smaller the values of *D_Mφ_* and *D_Nφ_*, the higher the similarity of the voltage waveforms. *D_WM_*(*d_M_*, *d_rM_*) and *D_WN_*(*d_N_*, *d_rN_*) reflect the overall distribution characteristics of the voltage waveforms, while *D_Mdtw_*(*d_M_*, *d_rM_*) and *D_Ndtw_*(*d_N_*, *d_rN_*) reflect the overall dynamic characteristics of the voltage waveforms. Additionally, *D_Mdtw_*(*d*′*_M_*, *d*′*_rM_*) and *D_Ndtw_*(*d*′*_N_*, *d*′*_rN_*) reflect the local dynamic characteristics of the voltage waveforms. *α*, *β*, and *γ* are the weights assigned to each distance measure, and they are based on the actual sampling accuracy requirements and the principle of entropy weighting, their respective values are set to 0.4, 0.3, and 0.3 [21].

## 3. System Fault Characteristics and Protection Process

### 3.1. Hybrid DC Multi-Infeed AC/DC Interconnected System Topology Structure

**Figure 1:**
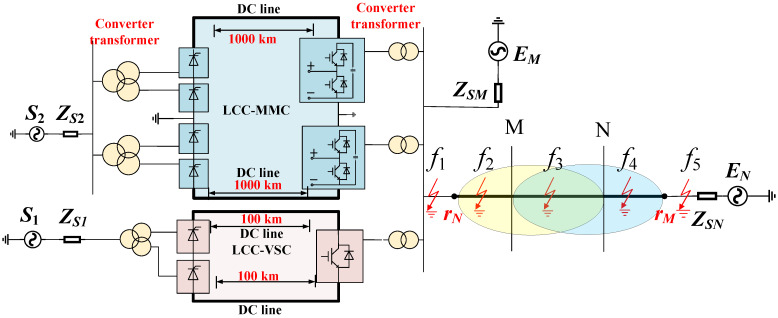
Topology diagram of the hybrid DC multi-feed system.

**Figure A1:**
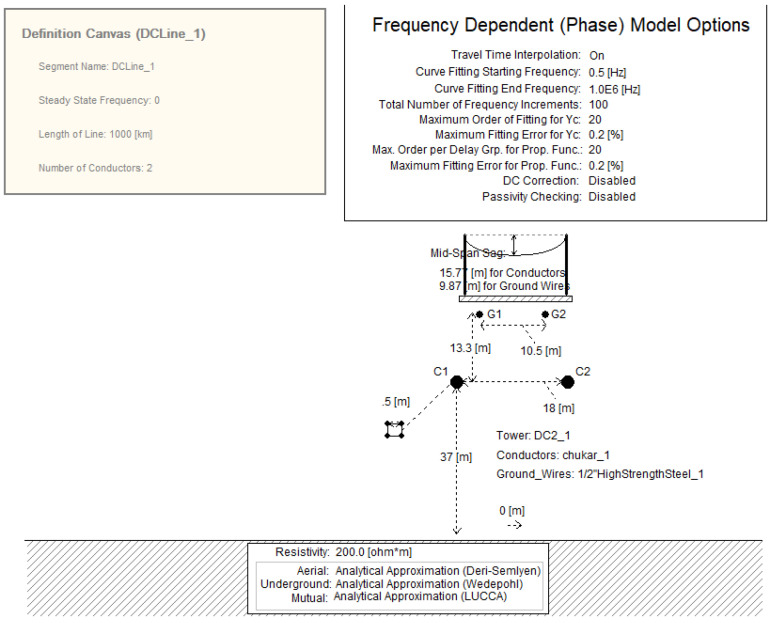
Frequency-domain equivalence model of DC transmission lines.

The constructed hybrid AC/DC interconnected system with multiple DC inputs, as shown in Figure 1, features the transmitting end’s DC section consisting of two distinct ±500 kV hybrid DC transmission lines: a bipolar double-end line commutated converter-modular multilevel converter (LCC-MMC) transmission system and a pseudo-bipolar double-end LCC-VSC transmission system. The power supply sides are represented by S_1_ and S_2_, with equivalent impedances of Z_S1_ and Z_S2_, respectively. The length of the DC line is shown in the figure, and the line model parameters are provided in the Figure A1 of the Appendix A. The vertical height of the tower is 37 m, and the number of conductors in the DC transmission line is 2. The receiving end’s AC section comprises a 230 kV dual-source system of E_M_ and E_N_, whose equivalent impedances are represented by Z_SM_ and Z_SN_, respectively, with M and N denoting the positions of the protection units on both sides of the line. *r_M_* represents the calculation reference point at end M, and *r_N_* represents the calculation reference point at end N, defining the protection scope from the protection installation point to the reference point on that side. The overlap of the protection scopes at both ends indicates the range for determining internal faults, denoted by the green area in Figure 1. This study focuses primarily on the central section, MN, where *f*_1_ and *f*_5_ represent faults occurring outside the protection scope, *f*_2_ represents a fault occurring solely within the protection scope at end N but outside the reverse area, *f*_3_ represents a fault occurring within the protection scope at both ends M and N, and *f*_4_ represents a fault occurring solely within the protection scope at end M but outside the forward area. The main model parameters are shown in Table 1.

In an LCC-VSC system, the rectifier side comprises two sets of twelve-pulse thyristor converters connected in series. The rectifier side direct current is input into the constant current control circuit, which controls the *α* angle to regulate the rectifier-side converters. On the inverter side, low-cost and highly stable two-level VSC modules are employed. Through vector control, the modulated waves are input into the pulse width modulation (PWM) controller, which then produces the control signals.

**Figure A2:**
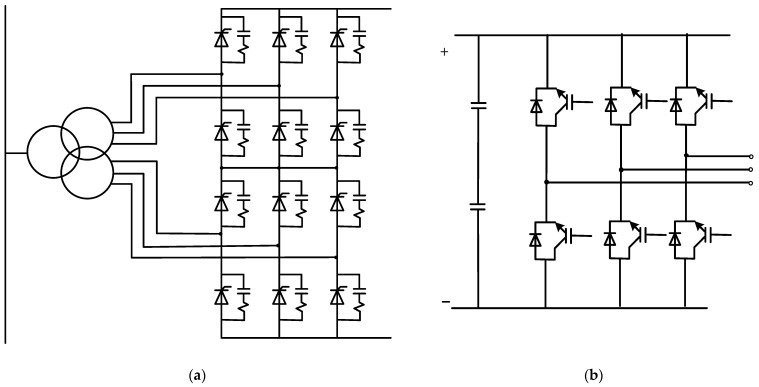

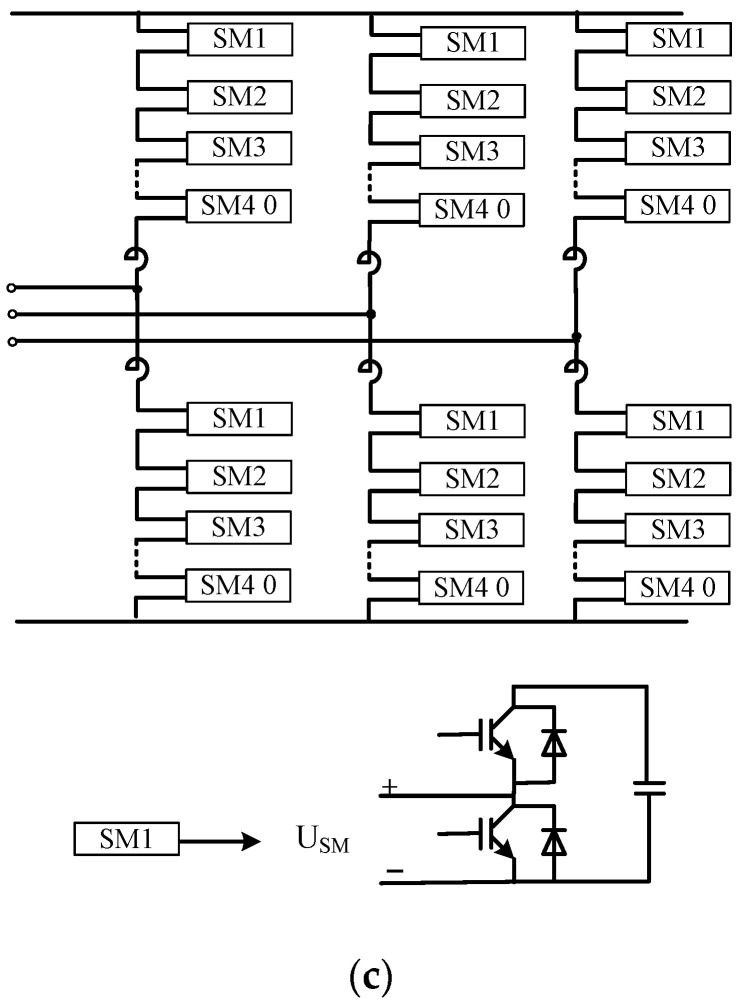
(**a**) Topology diagram of LCC converters; (**b**) topology diagram of VSC converters; (**c**) topology diagram of MMC converters.

In the LCC-MMC system, the rectification side also consists of two groups of twelve-pulse thyristor converters connected in series, and the control method is consistent with that of the LCC-VSC. The inverter side is a three-phase modular multilevel converter. In its topological diagram, the upper and lower bridge arms of each of the ABC three phases are composed of sub-modules SM connected in series. The power and current of the inverter side are input into the inner and outer loop control circuits, and the output signals are sent to the bridge arms to control the inverter side converter. Relevant topological diagrams are shown in the Figure A2 of the Appendix A.

### 3.2. Reference Voltage Calculation and System Fault Characteristics

The similarity of the voltage waveforms between the measuring point and the reference point is calculated to determine if a fault has occurred, and the position of the reference point needs to be determined. The reference point is usually located outside the protection line to ensure sufficient sensitivity. Additionally, to reduce errors, the reference point should not be too far away; scholars both domestically and abroad typically position it within the range of 1.2 to 2.0 times the length of the protected line [22]. After extensive simulation experiments, it has been found that placing the reference point at 1.3 times the length of the line can meet the experimental requirements outlined in this paper while allowing for flexible adjustments within the range based on specific protection needs and interference considerations.

Compared to traditional purely AC lines, in a multi-infeed interconnected grid, significant non-periodic components are introduced into the DC system after a fault occurs. This results in a more complex variation of electrical quantities in the AC lines. Obtaining accurate voltage phasors through traditional Fourier algorithms no longer guarantees accuracy. Instead, this paper proposes using instantaneous measurement methods to calculate the reference voltage, taking the M-side as an example.

**Figure 2:**
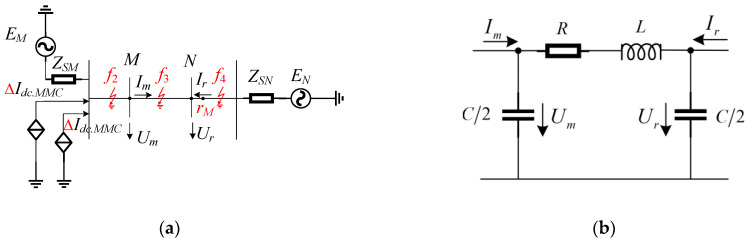
Schematic diagram of a system and line model (**a**) simplifying the system model; (**b**) schematic diagram of a system and line model.

In Figure 2a, the dual-power system consisting of E_M_ and E_N_ is depicted, with their respective equivalent impedances denoted as Z_SM_ and Z_SN_. Since the rectification side of the hybrid multi-infeed DC system employs constant current control, and the voltage on the inversion side is determined by the receiving-end AC bus voltage, both can be equivalently represented as voltage-controlled current sources controlled by the receiving end. ΔI_dc.MMC_ and ΔI_dc.VSC_ represent the equivalent fault component currents corresponding to the LCC-MMC and LCC-VSC hybrid DC systems, respectively. I_m_ and Um represent the measured instantaneous current and voltage values, while I_r_ and U_r_ represent the reference instantaneous current and voltage values. The notations *f*_2_, *f*_3_, and *f*_4_ indicate faults occurring in the reverse direction outside of the Mr_M_ zone, within the Mr_M_ zone, and in the forward direction outside of the Mr_M_ zone, respectively.

Based on the situation of the completed and ongoing multi-infeed AC/DC hybrid power grid projects, this paper sets a total line length of 160 km, using an equivalent π-type line model considering the electric shunt, as depicted in Figure 2b. After measuring the instantaneous current value *I_m_* and the instantaneous voltage *U_m_*, the reference voltage *U_r_* can be obtained through the differential equation in Equation (12).

(12) $$U_{r}=U_{m}-\left[R\left(I_{m}-\frac{C}{2}\frac{dU_{m}}{dt}\right)+L\frac{d\left(I_{m}-\frac{C}{2}\frac{dU_{m}}{dt}\right)}{dt}\right]$$

**Figure 3:**
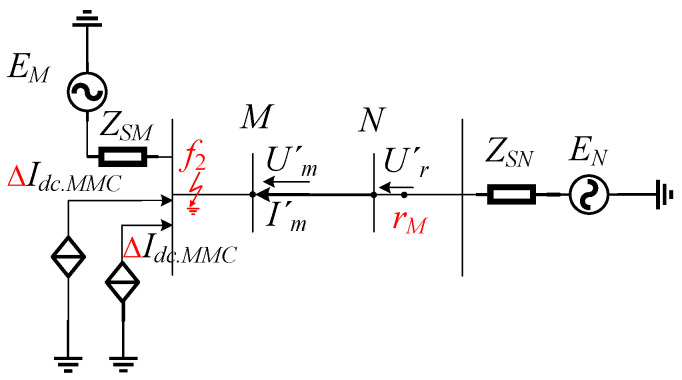
Schematic diagram of an out-of-reverse fault system.

Under normal operation, the voltage waveforms at the measurement point and reference point are similar. To analyze the fault characteristics of the system at different locations, we take the occurrence of a ground fault as an example and analyze the measured voltage *U_m_* and the reference voltage *U_r_*. We assume the fault occurs at location *f*_2_ as illustrated in Figure 2a, which represents an external fault in the opposite direction of *Mr_M_*, as shown in Figure 3.

At this point in time, the actual current direction is opposite to the measured current direction during normal operation, hence Equation (12) can be rewritten as follows:

(13) $$U_{m}^{\prime }=-\left[R_{f}^{\prime }\left(I_{m}^{\prime }-\frac{C_{f}^{\prime }}{2}\frac{dU_{m}^{\prime }}{dt}\right)+L_{f}^{\prime }\frac{d\left(I_{m}^{\prime }-\frac{C_{f}^{\prime }}{2}\frac{dU_{m}^{\prime }}{dt}\right)}{dt}\right]$$

(14) $$U_{r}^{\prime }=-\left[\begin{array}{l} I_{m}^{\prime }\left(R_{f}^{\prime }-R_{r}^{\prime }\right)-\frac{dU_{m}^{\prime }}{dt}\left(R_{f}\frac{C_{f}^{\prime }}{2}-R_{r}\frac{C_{r}^{\prime }}{2}\right)+\\ L_{f}^{\prime }\frac{d\left(I_{m}^{\prime }-\frac{C_{f}^{\prime }}{2}\frac{dU_{m}^{\prime }}{dt}\right)}{dt}-L_{r}^{\prime }\frac{d\left(I_{m}^{\prime }-\frac{C_{r}^{\prime }}{2}\frac{dU_{m}^{\prime }}{dt}\right)}{dt} \end{array}\right]$$

where *R*′*_f_*, *L*′*_f_*, and *C*′*_f_* represent the resistance, inductance, and capacitance values from measurement point *M* to fault point *k*_1_, respectively. *R*′*_r_*, *L*′*_r_*, and *C*′*_r_* represent the resistance, inductance, and capacitance values from measurement point *M* to reference point *r_M_*, respectively. By comparing the two equations, it can be concluded that although there are differences in the amplitude of the measured voltage *U*′*_m_* and the reference voltage *U*′*_r_*, their variation trends are consistent. The computational relationship deduced from Equation (14) is identical to that of Equation (12), indicating that when an external fault occurs in the reverse direction area, the measured voltage and the reference voltage waveforms are similar.

**Figure 4:**
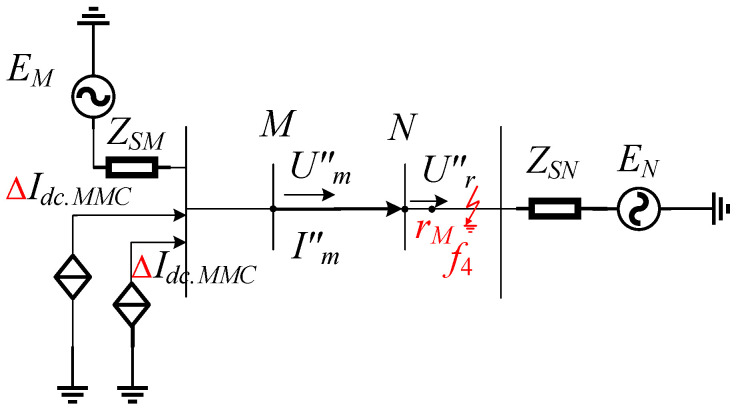
Schematic diagram of an out-of-zone fault system.

We assume that the fault occurs at position *f*_4_ as shown in Figure 2a, i.e., an external fault in the positive direction of *Mr_M_*, as illustrated in Figure 4.

At this time, the actual current direction is the same as the measured current direction under normal operation, rewriting Equation (12) as follows:

(15) $$U_{m}^{\prime \prime }=R_{m}^{\prime \prime }\left(I_{m}^{\prime \prime }-\frac{C_{f}^{\prime \prime }}{2}\frac{dU_{m}^{\prime \prime }}{dt}\right)+L_{f}^{\prime \prime }\frac{d\left(I_{m}^{\prime \prime }-\frac{C_{f}^{\prime \prime }}{2}\frac{dU_{m}^{\prime \prime }}{dt}\right)}{dt}$$

(16) $$U_{r}^{\prime \prime }=I_{m}^{\prime \prime }\left(R_{f}^{\prime \prime }-R_{r}^{\prime \prime }\right)-\frac{dU_{m}^{\prime \prime }}{dt}\left(R_{f}^{\prime \prime }\frac{C_{f}^{\prime \prime }}{2}-R_{r}^{\prime \prime }\frac{C_{f}^{\prime \prime }}{2}\right)+L_{f}^{\prime \prime }\frac{d\left(I_{m}^{\prime \prime }-\frac{C_{f}^{\prime \prime }}{2}\frac{dU_{m}^{\prime \prime }}{dt}\right)}{dt}-L_{r}^{\prime \prime }\frac{d\left(I_{m}^{\prime \prime }-\frac{C_{r}^{\prime \prime }}{2}\frac{dU_{m}^{\prime \prime }}{dt}\right)}{dt}$$

where *R*″*_f_*, *L*″*_f_*, and *C*″*_f_* represent the resistance, inductance, and capacitance values from measurement point *M* to fault point *k*_3_, respectively. *R*″*_r_*, *L*″*_r_*, and *C*″*_r_* respectively represent the resistance, inductance, and capacitance values from measurement point *M* to reference point *r_M_*. By comparing Equations (15) and (16), it can be observed that although there is a difference in the magnitude of the measured voltage *U*″*_m_* and the reference voltage *U*″*_r_*, their variation follows a consistent pattern. The computational relationship derived from Equation (16) is the same as Equation (12), indicating that when an external fault occurs, the measured voltage and the reference voltage waveforms are similar. 

**Figure 5:**
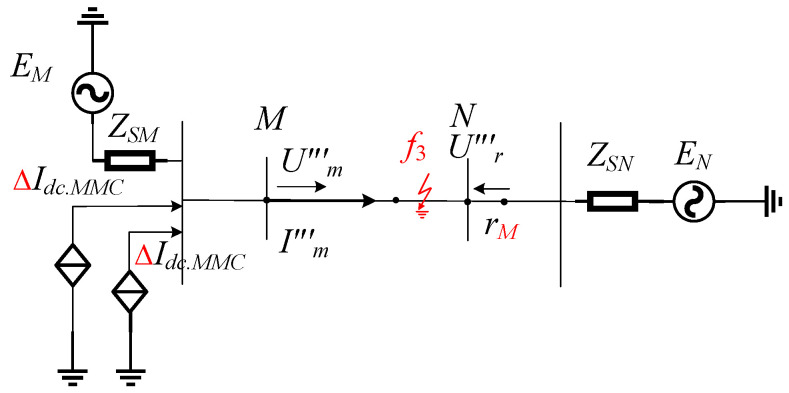
Schematic diagram of the fault system in the area.

We suppose that the fault has occurred at *f*_3_ in the area of *Mr_M_*, as shown in Figure 2a, depicting an internal fault in the *Mr_M_* area, as illustrated in Figure 5.

At this time, the fault branch between *M* and *r_M_* is increased, and the line structure is disrupted. Based on the characteristic that the distance from measuring point *M* to reference point *r_M_* is greater than to the fault point during an internal fault, Equation (12) is modified as follows:

(17) $$U_{m}^{\prime \prime \prime }=R_{f}^{\prime \prime \prime }\left(I_{m}^{\prime \prime \prime }-\frac{C_{f}^{\prime \prime \prime }}{2}\frac{dU_{m}^{\prime \prime \prime }}{dt}\right)+L_{f}^{\prime \prime \prime }\frac{d\left(I_{m}^{\prime \prime \prime }-\frac{C_{f}^{\prime \prime \prime }}{2}\frac{dU_{m}^{\prime \prime \prime }}{dt}\right)}{dt}$$

(18) $$\begin{array}{c} U_{r}^{\prime \prime \prime }=\left[R_{r}^{\prime \prime \prime }\left(I_{m}^{\prime \prime \prime }-\frac{C_{r}^{\prime \prime \prime }}{2}\frac{dU_{m}^{\prime \prime \prime }}{dt}\right)+L\frac{d\left(I_{m}^{\prime \prime \prime }-\frac{C_{r}^{\prime \prime \prime }}{2}\frac{dU_{m}^{\prime \prime \prime }}{dt}\right)}{dt}\right]-U_{m}^{\prime \prime \prime }\\ =\frac{dU_{m}^{\prime \prime \prime }}{dt}\left(R_{f}^{\prime \prime \prime }\frac{C_{f}^{\prime \prime \prime }}{2}-R_{r}^{\prime \prime \prime }\frac{C_{r}^{\prime \prime \prime }}{2}\right)-I_{m}^{\prime \prime \prime }\left(R_{f}^{\prime \prime \prime }-R_{r}^{\prime \prime \prime }\right)+\\ L_{f}^{\prime \prime \prime }\frac{d\left(\frac{C_{f}^{\prime \prime \prime }}{2}\frac{dU_{m}^{\prime \prime \prime }}{dt}-I_{m}^{\prime \prime \prime }\right)}{dt}-L_{r}^{\prime \prime \prime }\frac{d\left(\frac{C_{f}^{\prime \prime \prime }}{2}\frac{dU_{m}^{\prime \prime \prime }}{dt}-I_{m}^{\prime \prime \prime }\right)}{dt} \end{array}$$

where *R*‴*_f_*, *L*‴*_f_*, and *C*‴*_f_* represent the resistance value, inductance value, and capacitance value from measuring point *M* to fault point *k*_2_, respectively. *R*‴*_r_*, *L*‴*_r_*, and *C*‴*_r_* represent the resistance value, inductance value, and capacitance value from measuring point *M* to reference point *r_M_*. By comparing Equations (17) and (18), it can be inferred that although there are differences in the amplitude of the measured voltage *U*‴*_m_* and the reference voltage *U*‴*_r_*, their variation trends remain consistent. Equation (18) deduces an operational relationship opposite to that of Equation (12), indicating that when an internal fault occurs, the measured voltage and the reference voltage waveforms are dissimilar.

In summary, it can be concluded that during an internal fault, the measured voltage and the reference voltage waveforms are dissimilar, whereas during normal operation and external fault occurrence, the voltage waveforms are similar. This provides a theoretical basis for the subsequent use of voltage comprehensive waveform similarity.

### 3.3. Protection Action Criteria

By employing the calculation method in Section 2.5, the comprehensive waveform similarity at both ends is obtained. From the computed results for the three phases, the maximum values are denoted as MAX(*D_Mφ_*) and MAX(*D_Nφ_*), and a threshold value *D_set_* is established. When the voltage comprehensive waveform similarity exceeds the threshold value, it indicates a dissimilarity between the two voltage waveforms. Considering the topological structure and operational conditions of the AC/DC hybrid system, along with practical engineering requirements and a large volume of simulation data, the threshold value *D*_set_ is chosen as 1.2. By comparing the relationship between MAX(*D_Mφ_*), MAX(*D_Nφ_*), and the protection threshold value *D_set_*, fault locations can be further determined.

When MAX(*D_Mφ_*) < *D_set_* and MAX(*D_Nφ_*) > *D_set_*, the fault occurs between protection device *M* and reference point *r_M_*, that is, the position *f*_2_ in Figure 1 represents an external fault.

When MAX(*D_Mφ_*) > *D_set_* and MAX(*D_Nφ_*) < *D_set_*, the fault occurs between protection device *N* and the reference point *r_N_*, indicating that the fault location is external, as illustrated in position *f*_4_ in Figure 1.

When MAX(*D_Mφ_*) < *D_set_* and MAX(*D_Nφ_*) < *D_set_*, the fault occurs in areas outside protection devices *M* and *N*, i.e., positions *f*_1_ and *f*_5_ in Figure 1 indicate an external fault zone.

When MAX(*D_Mφ_*) > *D*_set_ and MAX(*D_Nφ_*) > *D_set_*, the fault is determined to have occurred between the two protective devices, i.e., the location *f*_3_ in Figure 1 indicates an internal fault, triggering the protective action.

When a fault occurs, the fault component of the current will increase or decrease. Based on the magnitude of this sudden change, protection initiation criteria can be established, and the fault component of the current can be extracted using the superposition theorem.

(19) $$\Delta I_{s}(t)=I_{s}(t)-I_{s}(t-T)$$

where *t* denotes the sampling instant. *I_s_*(*t*) represents the actual current value at location *s* at instant *t*. Δ*I_s_*(*t*) stands for the fault component of the current extracted at location *s* at instant *t* for protection measurement. *T* represents the selected pre-fault sampling instant. Considering the system’s operational stability, typically the two sampling cycles before the fault are chosen. Based on the variation in the fault component of the current obtained from Equation (19), the initiation criterion can be obtained as follows:

(20) $$\Delta I_{sM\varphi }>0.01kI_{n}$$

(21) $$\Delta I_{sN\varphi }>0.01kI_{n}$$

where Δ*I_sMφ_* and Δ*I_sNφ_* denote the change in current fault components at the protection installation points on sides *M* and *N*, respectively. Here, *φ* = A, B, C represents the phase sequence index, *k* is the reliability factor, and *I_n_* stands for the rated line current. As long as any of the judgment conditions in Equations (20) and (21) are satisfied, the calculation of the comprehensive voltage waveform distance similarity can be initiated to further determine the fault area.

**Figure 6:**
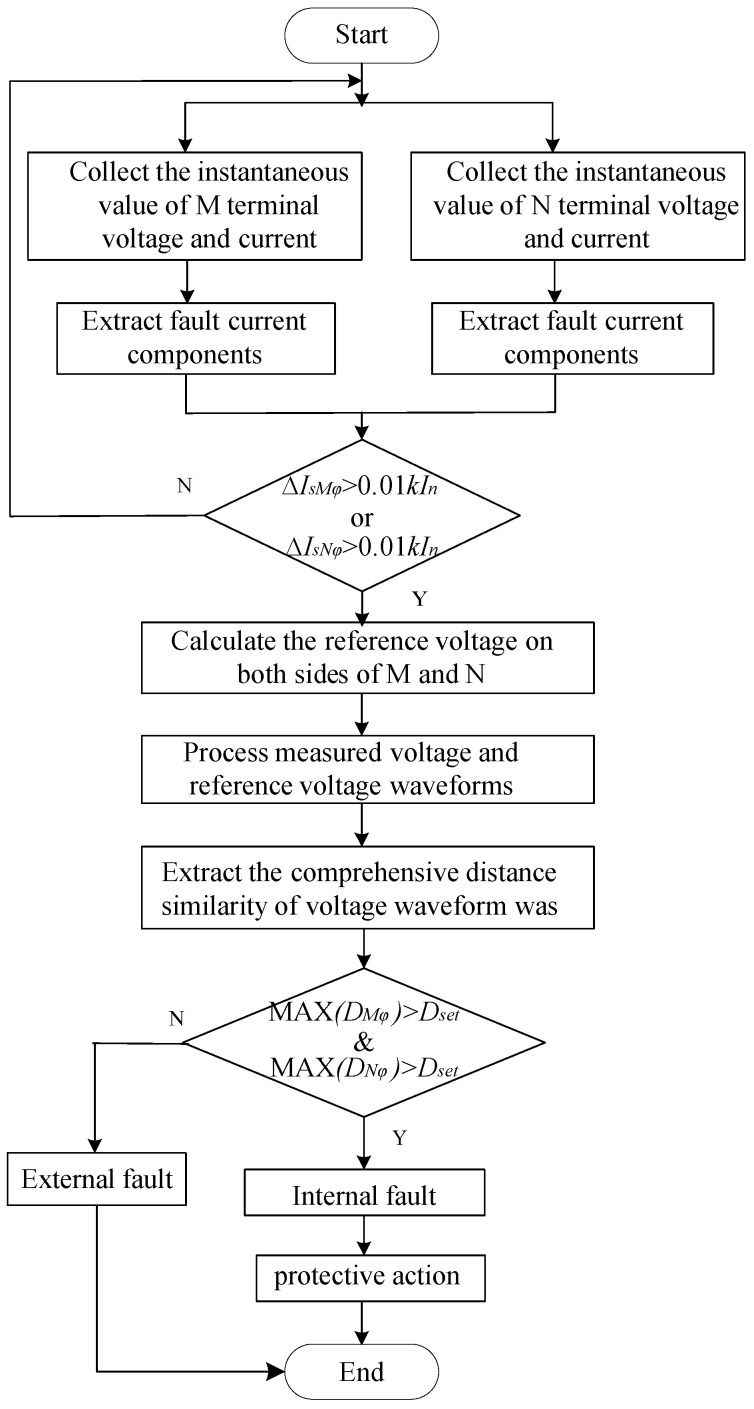
Protection flow chart.

Based on the above fault analysis process and the introduction of the protection scheme, the overall protection process can be illustrated as shown in Figure 6, with the specific steps outlined below:

(1) Run the system model, debug the protection measurement devices, collect electrical signal data, and extract the sampled values of voltage and current for the *M* and *N* ends of the AC transmission line using preset sensors.
(2) Extract transient changes in fault components from the sampled current values at ends *M* and *N* and determine if a fault has occurred using a protection initiation criterion. When the criterion is met, it indicates a fault in the system.
(3) Calculate the reference voltages at both ends based on the sampled voltage and current values and use the method of least squares to fit the waveform trend of the sampled and calculated values, thereby filtering out high-order harmonic interference.
(4) Derive the comprehensive waveform distance similarity metrics *D_Mφ_* and *D_Nφ_* between the measured voltage and the reference voltage at both ends of the line. Compare the maximum values of *D_Mφ_* and *D_Nφ_* with the protection set value *D_set_* to determine the fault occurrence region.

When *D_Mφ_* > *D_set_* and *D_Nφ_* > *D_set_*, the fault is determined to occur between the two protective devices, indicating an internal fault, located at position *f*_3_ in Figure 1; otherwise, it is determined to be an external fault.

## 4. Experimental Verification

### 4.1. Introduction to the Experimental Platform

To verify the theoretical analysis and the feasibility of the protection scheme mentioned above, the PSCAD/EMTDC simulation platform is utilized to construct the hybrid DC multi-infeed system, as depicted in Figure 1, for power system simulation. The electrical parameters are adjusted, and various fault occurrences are simulated under different conditions by controlling the fault area, line positions, and other interfering factors. Subsequently, the validation of the protection algorithm is carried out in MATLAB.

The sampling window for calculating the comprehensive voltage distance similarity in this paper is 1 ms, and the sampling frequency for line protection is 10 kHz. Therefore, fault detection requires only 10 sampling points. In practical engineering applications, the synchronization error of the commonly used global positioning system (GPS) synchronization method is less than 2 μs. The protection algorithm investigated in this paper calculates the similarity value within the sampling interval and compares them to find the maximum value, making it possible to accurately identify the fault position within a 1 ms delay, without strict bi-directional timing and communication synchronization.

In conclusion, the simulated duration in this paper is set as 3 s, and the fault occurs at 2.25 s and lasts for 0.05 s. Due to the rapid change in the current fault component, the protection can be activated in about 0.5 ms, while the simulation data collection and the calculation of comprehensive voltage distance similarity can be completed within 2 ms. Considering a certain margin, the proposed method can operate quickly and reliably within 3 ms.

### 4.2. In-Zone Fault Simulation Result

**Figure 7:**
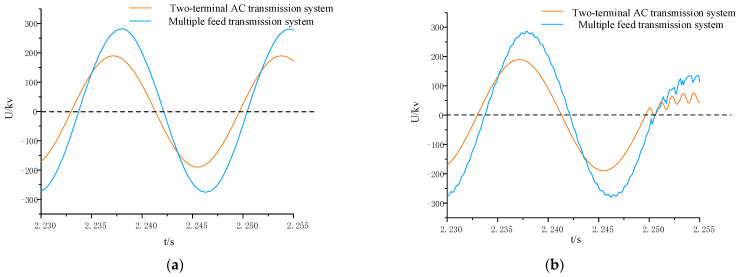
Comparison of measured voltage waveforms between a pure AC system and a hybrid multi-feed system. (**a**) Comparison of measured voltage waveforms under normal operation; (**b**) comparison of measured voltage waveforms under faults in the area.

In the positive direction of the AC line, a phase A-to-ground fault is set at a distance of 50 km. To stabilize the feed-in of the hybrid DC system, the fault is initiated at 2.25 s and lasts for 0.05 s. Taking the data at the M-end as an example, the simulation compares the waveform differences between the normal and fault states of both a pure AC system and a hybrid DC multi-feed system. The simulation window length is 0.025 s, and the simulation results are shown in Figure 7.

**Figure 8:**
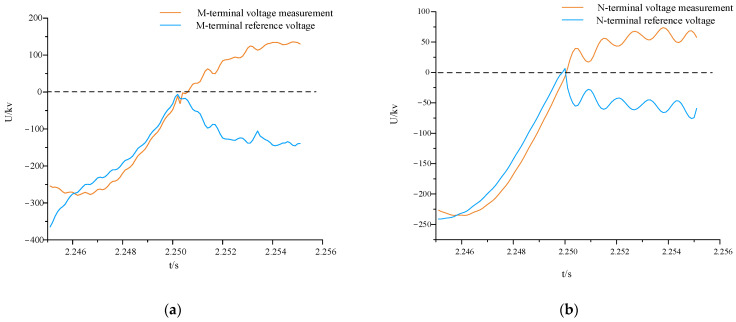
Comparison of measured voltage waveforms between a pure AC system and a hybrid multi-feed system. (**a**) Comparison of measured voltage waveforms under normal operation; (**b**) comparison of measured voltage waveforms under faults in area.

In Figure 7, it is evident that after the introduction of multi-feed-in, both the voltage amplitude and phase undergo changes. Additionally, the system, besides experiencing voltage reduction after the fault, is also influenced by higher harmonics, leading to irregular oscillations in the fault waveform. By extracting data from the N-end and computing the reference voltage at both ends, Figure 8 compares the measured voltage and reference voltage waveforms.

In Figure 8a,b, the measured voltage and reference voltage waveforms at terminals M and N both exhibit an upward trend before the fault occurs, with high waveform similarity. After the fault occurs, the measured voltage continues to rise, while the reference voltage decreases. The magnitudes of voltage fluctuations in both waveforms are quite similar, exhibiting an almost symmetrical relationship around 0 kV, albeit in opposite directions. This aligns with the conclusion drawn in Section 3.2.

**Figure 9:**
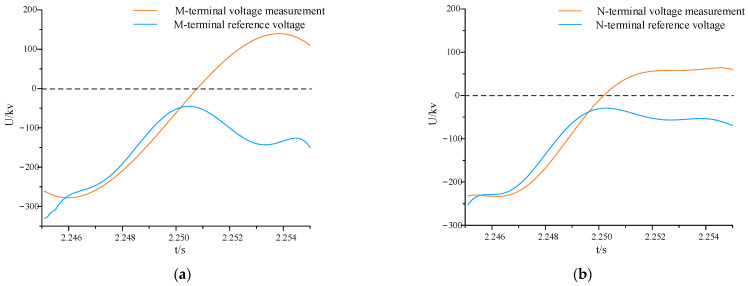
Comparison of voltage waveforms after treatment. (**a**) Comparison of M-terminal voltage waveforms after optimization; (**b**) comparison of N-terminal voltage waveforms after optimization.

For irregular variations in fault waveforms in mixed multi-feed systems, directly applying a similarity algorithm focusing on detailed levels can lead to significant errors. However, after applying the waveform processing method in Section 2.1, waveform optimization can be achieved while retaining trend information, as illustrated in Figure 9.

As shown in Figure 9, the processed waveform is smoother and largely retains the trend information of the waveform changes, reducing errors in the subsequent calculation of the comprehensive distance similarity of voltage waveforms.

To study the changes in the comprehensive distance similarity of internal fault waveforms under various operating conditions, faults were set at 50 km in the positive direction of lines M and N, with controlled transition resistance size. This involved simulating single-phase-to-ground faults (A-G), two-phase-to-ground faults (BC-G), three-phase-to-ground faults (ABC-G), and phase-to-phase short-circuit faults (BC). The fault initiation time was set at 2.25 s, with a duration of 0.05 s. Simulation data were tabulated in Table 2.

In Table 2, bolded data indicate values greater than the set value, and data marked in red, such as *D_MA_* = 4.4298 and *D_NA_* = 4.6376, represent the maximum values in this dataset. As Table 1 demonstrates, during the occurrence of faults, the comprehensive distance similarity of the faulted phase consistently exceeds the set value *D_set_*, enabling correct action and non-operation for non-faulted phases. All of these are in line with the simulation results and theoretical analysis, meeting the protection requirements.

### 4.3. Out-of-Zone Fault Simulation Result

**Figure 10:**
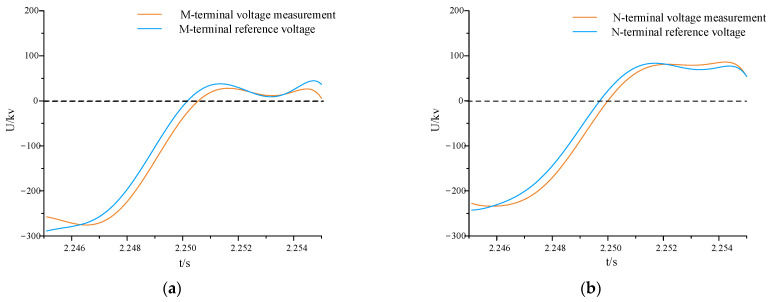
Comparison of measured voltage and reference voltage waveform during an out-of-zone fault. (**a**) Comparison of M-terminal voltage waveforms; (**b**) comparison of N-terminal voltage waveforms.

Referring to Figure 1, a single-phase bolted fault (*f*_1_) is established 40 km outside the M and N lines in the opposite direction. Voltage and current data from both ends of M and N are extracted, the reference voltage is computed, and waveforms are processed. A comparison of the measured voltages at both ends and the reference voltage waveform is shown in Figure 10.

In Figure 10, the trends in the measured voltage and the reference voltage waveform are consistent, aligning with the conclusion derived from theoretical analysis concerning the similarity of the two voltage waveforms during an external fault. To further investigate the comprehensive distance similarity characteristics of the voltage waveform during external faults under various operating conditions, with reference to Figure 1, *f*_1_ and *f*_2_ are faults set, respectively, 30 km and 10 km outside the opposite direction of the M and N lines, and *f*_4_ and *f*_5_ are faults set, respectively, 10 km and 30 km outside the forward direction of the M and N lines.

These faults simulate single-phase earth faults, two-phase earth faults, and three-phase earth faults. The fault initiation time is set at 2.25 s, with a duration of 0.05 s. The simulated data are summarized in Table 3.

Based on theoretical analysis, when an external fault occurs, the measured voltage and the reference voltage waveform are similar. In Table 3, bold data indicate values greater than the set value, which triggers the protective action. While red data represent maximum values within the dataset. For external faults occurring at *f*_1_ and *f*_5_, the data in Table 3 are all less than the set value (*D_set_*), thus neither end of the protection devices act falsely. In the case of an *f*_2_ external fault, as MAX(*D_Mφ_*) remains consistently less than the set value *D_set_*, both ends of the protection devices remain unaffected. Similarly, when an *f*_4_ external fault occurs, as MAX(*D_Nφ_*) consistently remains less than the set value *D_set_*, both ends of the protection devices remain unaffected. In conclusion, the simulation results for external faults meet the protection requirements.

### 4.4. Protection Action Status

**Figure 11:**
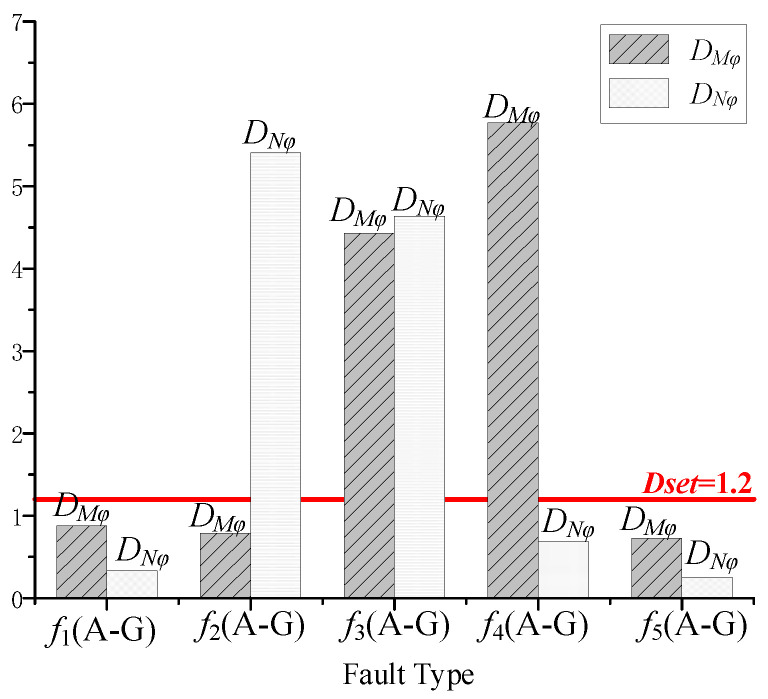
Simulation results for internal and external faults identification.

Currently, in the practical engineering of AC transmission lines, the highest proportion of faults is single-phase-to-ground faults. Taking the occurrence of single-phase high-impedance ground faults as an example, simulated protection data are illustrated in Figure 11.

From Figure 11, it can be visually observed that the operating condition of *D_Mφ_* > *D_set_* and *D_Nφ_* > *D_set_* is satisfied only when an internal fault *f*_3_ occurs. Selecting the setting value *D_set_* = 1.2 provides a certain margin for the calculation results when the waveforms are similar. The proposed method can identify internal and external faults reliably and can meet the protection requirements.

### 4.5. Anti-Interference Analysis

In actual engineering scenarios, there exist numerous interferences. To test the method’s resistance to interference, this paper introduced a 10 dB noise ratio interference under a 100 Ω grounding fault and simulated different positions within the zone. These positions were at 10%, 50%, and 90% of the total line length. The simulation data are summarized in Table 4:

In Table 4, bold data indicate values greater than the set value, which triggers the protective action. While red data represent maximum values within the dataset. When comparing the interference-added comprehensive distance similarity calculation results at the 50% total line length with the data in Table 2, it is observed that there are varying degrees of increase or decrease in results after the addition of noise interference. For the 10% and 90% total length positions, the comprehensive distance similarity of the fault phase is greater than the setting value *D_set_*, indicating non-rejection of misoperation. Conversely, the comprehensive distance similarity of the non-fault phase is lower than the setting value *D*_set_, demonstrating non-operation. This shows that the proposed method can correctly operate even in the presence of noise interference, meeting the requirements for protection.

### 4.6. Comparison and Discussion

To demonstrate the superiority of the proposed method, it is compared with [23] and the longitudinal differential protection scheme. Ref. [23] uses the conclusion that reference voltage and measured voltage waveforms have different characteristics during faults inside and outside the area to roughly determine the fault location through the Pearson correlation coefficient. Then, adopting the longitudinal protection logic, it provides a more accurate judgment result. However, Ref. [23] lacks an analysis of the protection scheme’s ability to withstand transition resistance. The longitudinal current differential protection scheme utilizes the assigned values and phases of both sides of the protected line to determine whether a fault has occurred. The criterion is shown in Equation (22).

(22) $$\dot{I_{d}}>\dot{{KI}_{r}}$$

where the differential current $\dot{I_{d}}=\left|{\dot{I}}_{M}+{\dot{I}}_{N}\right|$ and the braking current $\dot{I_{r}}=\left|{\dot{I}}_{M}{\dot{I}}_{N}\right|$ are defined. ${\dot{I}}_{M}and{\dot{I}}_{N}$ represent the measured currents at both ends of M and N, respectively. *K* is the braking coefficient, and the operating threshold is set as the maximum unbalanced current.

Regarding the limitations of the proposed method, a comparison section has been added to investigate the performance of various protection schemes in high-resistance grounding scenarios. Three methods were applied to a self-constructed experimental model. Single-phase grounding faults (A-G), two-phase grounding faults (BC-G), three-phase grounding faults, and inter-phase short-circuit faults were simulated at a distance of 50 km from the M-end. The faults occurred at 2.25 s and lasted for 0.05 s. Transition resistances of 100 Ω, 500 Ω, and 1000 Ω were set [24], respectively. Under the same system parameters and fault assumptions, the comparison results of the three schemes are shown in Table 5.

Since [23]’s sampling frequency is 4 kHz, it requires 25 sampling points with a data window of 6.25 ms, while the proposed method uses 10 sampling points with a data window of 1 ms. Therefore, Ref. [23] has a larger computational burden and slower speed. The current-based differential protection scheme acts at 4.6 ms under a 100 Ω transition resistor, while the proposed method acts at 3 ms. Therefore, the proposed method has a faster speed. By setting different transition resistances and simulating the protection action of the three methods under different transition resistances, it can be concluded that the proposed method can still operate correctly at 1000 Ω, indicating a stronger tolerance for transition resistance.

The structure of modern power grids is becoming increasingly complex, with more and more common scenarios such as multi-infeed, multi-port, and AC/DC hybrid transmission. This protection scheme provides a solution to ensure effective protection under various power grid structures. On the other hand, the scheme can accurately identify the area where a line fault occurs, effectively preventing fault propagation and improving the overall stability of the power grid. At the same time, fault diagnosis is performed through comprehensive distance similarity, ensuring correct operation under different transition resistances and noise interference, satisfying the selectivity, speed, sensitivity, and reliability of relay protection.

In this paper, a sampling frequency of 10 kHz is set, resulting in a large amount of sampled data. The similarity of waveforms is characterized and calculated using both Euclidean distance and Dynamic Time Warping (DTW) distance. The implementation of this method requires a certain amount of computational resources and has high requirements for microprocessors (such as CPU, memory, and GPU). This may limit the application of the proposed method in practical engineering, and further research is needed.

## 5. Conclusions

In the context of hybrid AC/DC systems, the mutual coupling between AC and DC components can affect the performance of AC-side protection. Aiming at the problem, this article proposes a longitudinal protection scheme that relies on voltage waveform similarity based on an analysis of the voltage fault characteristics of hybrid AC/DC multi-infeed receiving-end AC lines. Through simulation validation, the conclusions can be drawn as follows:

(1) On one hand, the use of Euclidean distance and Dynamic Time Warping (DTW) distance to characterize waveform similarity avoids the impact of data desynchronization and potential misoperation or refusal to operate caused by relying solely on a single distance metric. On the other hand, the use of least squares fitting for voltage waveforms effectively mitigates the interference from higher-order harmonics.
(2) The proposed longitudinal protection scheme based on voltage waveform similarity has been validated through simulations, demonstrating correct operation under various fault distances, fault types, transition resistances, and noise disturbances. Compared with other protection schemes, this approach offers faster and more reliable operation within 3 ms, improves speed and adaptability, and is not affected by data desynchronization, thus meeting protection requirements.
(3) The proposed scheme in this article is built upon an analysis of fault characteristics in multi-infeed AC/DC systems, providing theoretical support for fault protection research in all types of hybrid AC/DC interconnected systems under various fault conditions.

## Figures and Tables

**Table 1:** Model parameters.

Parameters	Numerical Value
Terminal voltage E_M_/E_N_ (/kV)	230
Short-circuit ratio	5
MN line length (/km)	100
Two-terminal out-of-zone line length (/km)	30
Total length at receiving end (/km)	160
Positive-sequence parameters of line R_1_ (Ω/km)	0.01782
Positive-sequence parameters of line ωL_1_ (Ω/km)	0.3139
Positive-sequence parameters of line ωC_1_ (S/km)	3.626 × 10^−6^
Zero-sequence parameters of line R_0_ (Ω/km)	0.2952
Zero-sequence parameters of line ωL_0_ (Ω/km)	1.039
Zero-sequence parameters of line ωC_0_ (S/km)	2.414 × 10^−6^

**Table 2:** Comprehensive distance similarity of fault waveforms in area *D_MA_*.

Fault Type	Transition Resistance (Ω)	*D_MA_*	*D_MB_*	*D_MC_*	*D_NA_*	*D_NB_*	*D_NC_*
Single-phase ground fault (AG)	0.01	4.4298	0.3175	0.8093	4.6376	0.2579	0.2792
	50	3.1682	0.3248	0.7994	3.2402	0.2459	0.2690
	100	2.7807	0.3268	0.7998	2.6619	0.2279	0.2612
	500	**1.7382**	0.3553	0.4257	**2.045**	0.2027	0.2303
	1000	**1.7436**	0.3512	0.4125	**2.0861**	0.2053	0.2339
Double-phase ground fault (BCG)	0.01	0.1387	3.3960	**2.5062**	0.4000	7.5495	**4.4422**
	50	0.4916	6.4778	**3.7474**	0.1258	7.6393	**4.1362**
	100	0.5539	6.5028	**4.8863**	0.1434	7.7454	**4.1295**
	500	0.4442	**6.9463**	**4.5904**	0.1367	**7.0183**	**3.9651**
	1000	0.4219	**6.8553**	**4.6241**	0.1094	**7.1028**	**3.9552**
Three-phase ground fault (ABCG)	0.01	**5.2445**	8.6719	**3.0781**	**6.3565**	**5.2792**	6.3929
	50	**4.2174**	8.2139	**3.0262**	**4.4252**	**4.1751**	6.8504
	100	3.6431	**3.5486**	**2.8565**	**3.5486**	7.6422	**6.8159**
	500	**2.3411**	**4.1703**	**3.1288**	**2.0772**	**5.9005**	**4.9442**
	1000	**2.2115**	**4.7501**	**3.7163**	**2.0173**	**5.4799**	**3.9673**
Phase-to-phase short circuit fault (BC)		0.4916	7.4778	**4.8882**	0.1258	6.6393	**4.1362**

**Table 3:** Comprehensive distance similarity of an out-of-zone fault waveform.

Fault Location	Fault Type	*D_MA_*	*D_MB_*	*D_MC_*	*D_NA_*	*D_NB_*	*D_NC_*
*f*_1_	(A-G)	0.4191	0.3818	0.8793	0.2577	0.2016	0.3326
	(BC-G)	0.3387	0.7960	0.4305	0.3298	0.6940	0.4132
	(ABC-G)	0.5454	0.5267	0.3940	0.6781	0.8451	0.6219
*f*_2_	(A-G)	0.4237	0.3780	0.8514	**8.2461**	0.2011	0.3291
	(BC-G)	0.3398	0.7862	0.4297	0.3127	**4.6844**	**5.4057**
	(ABC-G)	0.5401	0.5227	0.3899	**4.6774**	**5.8057**	**4.6211**
*f*_4_	(A-G)	**3.4668**	0.3669	0.7471	0.1971	0.2014	0.3279
	(BC-G)	0.3598	**5.7681**	**4.4179**	0.3177	0.6873	0.4098
	**(** **ABC-G** **)**	**4.5129**	**4.5115**	**4.4187**	0.6228	0.8047	0.6047
*f*_5_	**(** **A-G** **)**	0.4795	0.3215	0.7258	0.0904	0.1995	0.2525
	**(** **BC-G** **)**	0.3671	0.7543	0.3938	0.1097	0.6574	0.3561
	**(** **ABC-G** **)**	0.4911	0.4981	0.3477	0.6129	0.7987	0.5917

**Table 4:** Simulation results of anti-jamming ability.

Fault Location	Fault Type	*D* *_MA_*	*D* *_MB_*	*D* *_MC_*	*D* *_NA_*	*D* *_NB_*	*D* *_NC_*
10%	(A-G)	**6.7142**	0.2533	0.0574	**5.7713**	0.2314	0.2732
	(BC-G)	0.4351	**4.4981**	**4.3163**	0.1473	**6.8017**	**4.1781**
	(ABC-G)	**7.0384**	**6.9961**	**6.6941**	**7.4620**	**8.0194**	**6.9814**
50%	(A-G)	**4.5695**	0.2654	0.7600	5.1755	0.2522	0.2696
	(BC-G)	0.4101	**3.4312**	**3.6343**	0.1347	**7.2423**	**3.8155**
	(ABC-G)	**4.8054**	**4.5203**	**3.3509**	**5.4671**	**6.2985**	**6.5022**
90%	(A-G)	**1.9995**	0.2467	0.7250	7.2727	0.1836	0.2470
	(BC-G)	0.2174	**3.3058**	**3.1673**	0.0571	**3.1982**	**2.0070**
	(ABC-G)	**4.3491**	**4.1847**	**2.7319**	**5.4894**	**6.7844**	**6.0876**

**Table 5:** Execution results of different models.

Model		[23]	Longitudinal Differential Protection	Ours
Sampling frequency		4 kHz	4 kHz	10 kHz
Data window		6.25 ms	1 ms	1 ms
Protection operation time		8 ms	4.6 ms	3 ms
The AG protection action situation	100 Ω	no	yes	yes
	500 Ω	no	no	yes
	1000 Ω	no	no	yes
The ABG protection action situation	100 Ω	no	yes	yes
	500 Ω	no	no	yes
	1000 Ω	no	no	yes
The ABCG protection action situation	100 Ω	no	yes	yes
	500 Ω	no	no	yes
	1000 Ω	no	no	yes

## Data Availability

Data are contained within the article.
